# Prdm8 Regulates the Morphological Transition at Multipolar Phase during Neocortical Development

**DOI:** 10.1371/journal.pone.0086356

**Published:** 2014-01-29

**Authors:** Mayuko Inoue, Takao Kuroda, Aya Honda, Mariko Komabayashi-Suzuki, Tae Komai, Yoichi Shinkai, Ken-ichi Mizutani

**Affiliations:** 1 Laboratory of Neural Differentiation, Graduate School of Brain Science, Doshisha University, Kyoto, Japan; 2 Department of Molecular and Cellular Biology, Graduate School of Biostudies, Kyoto University, Kyoto, Japan; 3 Cellular Memory Laboratory, RIKEN, Saitama, Japan; 4 Japan Science and Technology Agency, PRESTO, Tokyo, Japan; Osaka University Graduate School of Medicine, Japan

## Abstract

Here, we found that the PR domain protein Prdm8 serves as a key regulator of the length of the multipolar phase by controlling the timing of morphological transition. We used a mouse line with expression of *Prdm8*-mVenus reporter and found that Prdm8 is predominantly expressed in the middle and upper intermediate zone during both the late and terminal multipolar phases. Prdm8 expression was almost coincident with Unc5D expression, a marker for the late multipolar phase, although the expression of Unc5D was found to be gradually down-regulated to the point at which mVenus expression was gradually up-regulated. This expression pattern suggests the possible involvement of Prdm8 in the control of the late and terminal multipolar phases, which controls the timing for morphological transition. To test this hypothesis, we performed gain- and loss-of-function analysis of neocortical development by using in utero electroporation. We found that the knockdown of Prdm8 results in premature change from multipolar to bipolar morphology, whereas the overexpression of Prdm8 maintained the multipolar morphology. Additionally, the postnatal analysis showed that the Prdm8 knockdown stimulated the number of early born neurons, and differentiated neurons located more deeply in the neocortex, however, majority of those cells could not acquire molecular features consistent with laminar location. Furthermore, we found the candidate genes that were predominantly utilized in both the late and terminal multipolar phases, and these candidate genes included those encoding for guidance molecules. In addition, we also found that the expression level of these guidance molecules was inhibited by the introduction of the Prdm8 expression vector. These results indicate that the Prdm8-mediated regulation of morphological changes that normally occur during the late and terminal multipolar phases plays an important role in neocortical development.

## Introduction

**Figure 1:**
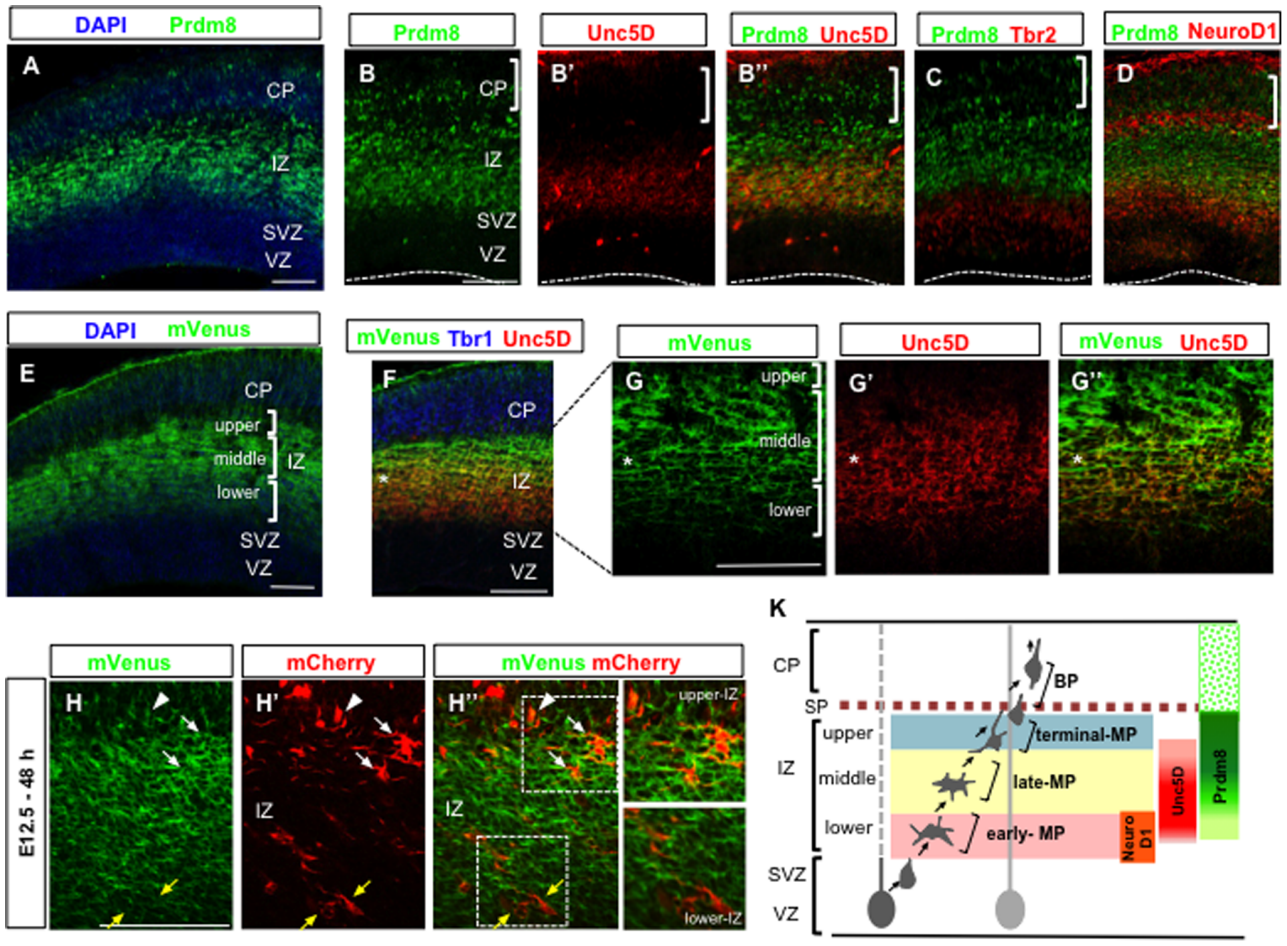
Prdm8 expression is mainly restricted to the middle- and upper -IZ during neocortical development. Immunostaining reveals that Prdm8 is weakly expressed in the lower-IZ (A, B). Prdm8 is highly expressed in the middle-IZ and upper-IZ, where its expression almost overlaps with Unc5d expression (B′, B″), which is a marker for late-MP phase, at E15.5. Prdm8-positive cells do not overlap with the Tbr2-positive cells (C), and some Prdm8-positive cells in the lower-IZ are positive for NeuroD1 (D). The mVenus expression pattern in transgenic mouse (E) is similar to the immunostaining pattern obtained with anti-Prdm8 antibody at E15.5 (See also Figure S1A–D, D′). The upregulation of Prdm8 (indicated by the asterisk) occurs in the MP cells at the late-MP phase (F, G, G′, G″). For improved visualization of the cell morphology during the MP phase, the *Prdm8*-mVenus embryo was electroporated at E12.5 with the CAG-mCherry vector, and analyzed 48 h later. Depending on their position within the IZ, the mCherry-positive MP or BP cells (white or yellow arrows; MP cells in the upper-IZ or lower-IZ, respectively. White arrowheads; BP cells in the upper-IZ) exhibit distinct levels of Prdm8 expression (H, H′, H″). Schematic drawing of the sequential expression of NeuroD1, Unc5D, and Prdm8 that coincides with the process of early post-mitotic differentiation in the neocortical development (K). The nuclei are stained with DAPI in A and E. Scale bars: 100 µm.

Most neocortical projection neurons originate from the asymmetric division of the radial glia progenitors in the ventricular zone (VZ) [1]. The neurons thus generated in VZ then move radially to the subventricular zone (SVZ) and lower-intermediate zone (IZ), where they assume a multipolar (MP) morphology (Figure 1K) [2], [3]. They also dynamically extend and retract multiple long projections, and move in apparently random directions towards both the lower-IZ and middle-IZ [4], [5]. Once the neurons move to the upper part of the IZ (upper-IZ), their morphology changes again from MP to bipolar (BP) [6]. Neurons with BP cell morphology move by locomotion along the radially oriented glial processes to their appropriate location within the developing cortical plate (CP) [7]. In these processes, transcriptional factors such as *Pax6*, *Tbr2*, *NeuroD1*, and *Tbr1* [8]–[12] and their gene regulatory networks play key roles to proceed cell state during neural migration from the VZ to the CP.

The molecular mechanisms that directly and/or indirectly control the “MP phase” in the IZ of neuronal migration are now being recognized [13]–[24]. For example, one recent study has shown that dynamic *FoxG1* expression is necessary for post-mitotic MP cells expressing *NeuroD1* to initiate *Uncd5D* expression and to rapidly proceed from the early (early-MP) to the late MP (late-MP) phase [13]. Another study indicated that neuronal migration mediated by EphrinA-EphA signaling, which possibly occurs during the MP phase, is essential for the proper intermixing of the neuronal types in the cortical column [14]. In addition, RP58 has been shown to control the MP-to-BP transition at terminal MP (terminal-MP) phase via the suppression of the neurogenin2-Rnd2 pathway [15], [16]. However, the importance of this MP phase for the establishment of mature cortical cytoarchitecture and the precise genetic control of this phase remain largely unknown.

Members of the recently described PRD1-BF1 and RIZ homology domain -containing (PRDM) proto-oncogene transcription factor family are new candidates implicated in the control of the developing central nervous system (CNS). This is because multiple genes in the Prdm family, including *Prdm8*, *Prdm12*, *Prdm13*, and *Prdm16* are expressed in the developing mouse CNS in a spatially and temporally restricted manner [25], [26]. These factors were originally identified as loci involved in cancer formation, and are also known to define cell fate [27], [28]. Moreover, a recent study has shown that Prdm8 is an obligate partner of Bhlhb5, with which it forms a repressor complex that directs neural circuit assembly [29].

Our previous study has shown that Prdm8 expression is tightly regulated in a spatio-temporal manner in the developing retina, spinal cord, and telencephalon [30]. In this study, we hypothesized that the specific expression pattern of Prdm8 in the late-MP and/or terminal-MP phases involves the regulation of the morphological changes that control the timing of neural differentiation. Accordingly, we aimed to elucidate the role of Prdm8 in the MP phase during neocortical development. In addition, to clarify the gene expression profiles in both the late-MP and terminal-MP phases, we analyzed sorted mVenus-positive cells by taking advantage of the specific expression pattern in the middle-IZ and upper-IZ of the mouse line of *Prdm8*-mVenus expression.

## Materials and Methods

### Ethics Statement

This study was carried out in strict accordance with the recommendations in the Guide for the Care and Use of Laboratory Animals of the Doshisha University. The protocol was approved by the Committee on the Ethics of Animal Experiments of the Doshisha University (Permit Number: 1339). All surgery was performed under sodium pentobarbital anesthesia, and all efforts were made to minimize suffering.

### Establishment of Prdm8 reporter and knockout mice

ICR or C57BL/6 mice were obtained from Shimizu Laboratory Supplies (Kyoto, Japan). For the generation of *Prdm8*-mVenus transgenic mice, BAC (bacterial artificial chromosome) bearing Prdm8 genomic locus of the C57BL/6 background was purchased from BACPAC Resource Center (Children's Hospital Oakland Research Institute, Clone ID: RP23-463H17). For construction of *Prdm8*-mVenus transgenes, the I_gk_ leader sequence following membrane-targeted Venus (mVenus) was recombined after the initial in-frame ATG of the exon 2 of the *Prdm8* gene by Red/ET Recombination (Figure S1) as previously described [31]. For the generation of *Prdm8* complete knockout mice (*Prdm8−/−*), both exon 2 and downstream of exon 5 of *Prdm8* were replaced by a loxP-flanked PGK-driven neomycin (Neo) and FRT-flanked PGK-driven Neo genes, respectively. After the treatment with Adeno-Cre, the clone, which was deleted Neo resistant gene, were selected. This targeted allele between exon 2 and downstream of exon 5 was later removed by crossing with *VASA*-Cre mouse line. Polymerase chain reaction (PCR) genotyping for the variants of *Prdm8* mutant loci was carried out using the following primer sets (Figure S3). p1: 5′-ACAAAAGCCCTACCTTCGCCCC-3′ p2: 5′-TAGAGCTTGCGGAACCCTTC-3′

### Plasmid construction

Full-length Prdm8 cDNA was amplified by PCR from cDNA clone (ID: 96300445H02) obtained from FANTOM (Functional Annotation of the mouse) using Takara LA Taq DNA polymerase (Takara) with the following primers (FW: 5′-CGGAATTCGATGGAGGATTCAGGCATCCA-3′, RV: 5′-CGGAATTCTTAATTATGCGAGGTCATGT-3′). For the generation of expression plasmid, amplified Prdm8 cDNA was inserted into pCAG-FLAG-IRES (pCAG-Prdm8). To generate mouse Prdm8 shRNA, the following hairpins was cloned into a pSUPER.retro.Puro vector, pPrdm8sh#629: 5′-AAAAAAATGTGTAAGGAGACGACCCGTTGACAGGAAGGGGTCGTCTCCTTACACATGCG-3′, and pPrdm8sh#2290: 5′-AAAAAAATTATGCGAGGTCATG TGCCGTGACAGGAAGGCACATGACCTCCCATAATTAG-3′. These hairpin-type shRNA template oligonucleotides were cloned into EcoRI site, and Puro vectors were linearized with EcoRI. The knockdown efficiency of constructed both plasmid in the primary neural progenitors was confirmed by the Western blotting, and its efficiency by pPrdm8sh#629 is much stronger than that by pPrdm8sh#2290. In addition, it should be noted that such a knockdown effect by both pPrdm8sh#629 and pPrdm8sh#2290 was specific to Prdm8 expression since no phenotype could be identified in the Prdm5 expression level, another transcription factor belonging to the Prdm family.

The pCAG-Unc5D and loxP-polyA-loxP (FloxP) conditional expression vector (pCAG-FloxP-EGFP-N1 and pCAG-Cre) were kindly provided by Drs. N. Yamamoto [32] and A. Shitamukai [33], respectively.

### In Utero Electroporation

Pregnant dams from wild-type (WT) ICR, *Prdm8*-mVenus, or *Prdm8*−/− mice were anesthetized by intraperitoneal injection with pentobarbital. Two uL of a mixture of plasmid DNA, which include 2.5 mg/mL target plasmid, 0.8 mg/mL reporter plasmid, and 2 mg/mL Fast Green was directly injected into the lateral ventricles of the embryonic forebrain by using a glass micropipette. In the case of conditional expression vector, a mixture of plasmid, which include 2.5 mg/mL target plasmid, 1 mg/mL pCAG-FloxP-EGFP-N1, 0.3 µg/mL pCAG-Cre, and 2 mg/mL Fast Green was used. The electroporation was performed using an electroporator (CUY21E, Nepa Gene) as previously described [34], [35].

### Immunostaining

Embryos were dissected, and the brains were fixed in 4% paraformaldehyde (PFA) for 1–3.5 hr. For postnatal stage, brains were fixed with 4%PFA overnight. Following 30% sucrose replacement, fixed brains were embedded in optimum cutting temperature (OCT) compound, and 20 micrometer slices were cut on a cryostat. The antibodies used were, rat anti-GFP (1∶500; nakalai tesk), rabbit anti-GFP (1∶200; IBL), rabbit anti-DsRed (1∶500; Invitrogen), goat anti-Unc5D (1∶100; R&D), rabbit anti-Tbr2 (1∶300; abcam), goat anti-NeuroD1 (1∶100; Santa Cruz), mouse anti-PCNA (1∶100; Cell Signaling), mouse anti-Tuj1 (1∶500; SIGMA), rabbit anti-Tbr1 (1∶100; abcam), mouse anti-RORb (1∶100; PERSEUS PROTEOMICS), rat anti-Ctip2 (1∶300; abcam), goat anti-Brn2 (1∶100; Santa Cruz), and mouse anti-Prdm8 [24]. Alexa Fluor-conjugated secondary antibodies (Invitrogen) were also used. EdU labeling (intraperitoneal injection of 12.5 mg/kg EdU) and staining were performed according to manufacturer's instructions (Invitrogen). Stainings were examined with Zeiss LSM 710 or Olympus IX81, and the images were finally processed with Adobe Photoshop.

### Cell Culture and in vitro electroporation

Primary embryonic neocortical cells were isolated from E14.5 WT mice, followed by TrypLE Express (Gibco) treatment and trituration to generate a single cell suspension. And, sixteen ug plasmid DNA was introduced into primary neocortical cells using Neon Transfection System (Life Technologies). Then, neocortical neurospheres were cultured for 2 days in serum-free media containing B27 without vitamin A (Gibco), N2 supplement (Gibco) and 10 ng/mL basic FGF.

### Quantitative real-time PCR

Quantitative real-time PCR was performed using SYBR green labeling (SYBR Premix Ex TaqII, Takara) and a TP850 Real-Time PCR System (Takara). Primer sequences used are available from the authors on request. GAPDH expression was used to normalize the samples, and each sample was run in triplicate.

### FACS analysis

Fluorescence-activated cell sorting (FACS) was performed using FACSAria II, and analyzed using FACSDiva 6.1 software (Becton Dickinson). The sorted cells were collected in the TRIzol (Life Technology).

### DNA microarray analysis

Total RNA was prepared using a RNeasy Mini kit (QIAGEN), and the quality was assessed with a BioPhotometer plus (Eppendorf). The cDNA synthesis and cRNA-labeling reactions were performed using the 3′IVT-Express Kit according to manufacturer's instructions (Affymetrix). High-density oligonucleotide arrays for Mus Musculus (Mouse Genome 430 2.0), containing 39,000 probes, were performed according to the Expression Analysis Technical Manual (Affymetrix).

### Statistics analysis

Statistical analysis was performed by using the Microsoft Excel. Student t test as stated in the appropriate experiments was used to test the significance. p<0.05 were considered statistically significant. Error bars are the SD.

## Results

### Prdm8 is expressed in the MP phase at embryonic stages

In the P19 embryonal carcinoma cell line, we found a gradual increase in the Prdm8 mRNA level with neural differentiation after the treatment with retinoic acid, and this increment was almost coincident with the expression of post-mitotic neuronal markers (data not shown). Indeed, other studies, including our previous study [29], [30] have shown that Prdm8 expression was restricted to post-mitotic cells from embryonic day 13 (E13) to E18 during neocortical development. However, the expression pattern of Prdm8 at each step of the process for the neural differentiation in the neocortex has not yet been fully elucidated. This prompted us to analyze the detailed expression pattern prevalent during neural differentiation by using each molecular marker, such as Pax6, Tbr2, NeuroD1, Unc5D, and Tbr1. By comparing the expression of Prdm8 at E15.5 (Figure 1A) to that of other transcription factors within the VZ, SVZ, lower-IZ and middle-IZ, subplate (SP), and CP, we found that the timing of Prdm8 expression was almost the same as that of Unc5D expression, which has been reported to be exclusive to the late-MP phase (Figure 1B, B′, B″) [5], [13], although Prdm8 expressed strongly in the upper-IZ and Unc5D expressed strongly in the middle-IZ, respectively. The other molecular markers used for this comparison include Pax6 (a marker for VZ [8]), Tbr2 (a marker for SVZ [8], [11]), NeuroD1 (a marker for early-MP phase [9], [13]), and Tbr1 (a marker for SP or deep-layer CP [8]). We noted a complementary expression pattern for Prdm8 and Tbr2 in the SVZ (Figure 1C), and found that Prdm8-positive cells were negative for Pax6 and Tbr1 expression at this stage (data not shown). In addition, Prdm8-positive cells at the early-MP phase were partially colocalized with those positive for NeuroD1 expression at the early-MP phase (Figure 1D).

Furthermore, we established the Prdm8 reporter mouse line, *Prdm8*-mVenus (Figure S1A), and observed that mVenus is strongly expressed in the post-mitotic cells of the developing neocortex (Figure S1C, D, D′), especially, from E13.5 to E15.5 (Figure S1E–G). This finding was consistent with the immunostaining data obtained with the Prdm8 antibody (Figure S1B, B′, B″). We also confirmed that the expression of mVenus at E15.5 was mainly restricted to both the late-MP and terminal-MP phases in the IZ (Figure 1E–G). Interestingly, Unc5D expression was found to be gradually downregulated to the point at which mVenus expression was gradually upregulated (indicated by the asterisk in Figure 1F, G, G′, G″). In addition, we introduced a CAG-promoter-driven mCherry-expressing vector into the ventricular surface of the lateral neocortex of an E12.5 *Prdm8*-mVenus embryo by using in utero electroporation [34], [35]. To confirm the correlation between the expression level of Prdm8 and the MP phase, the brain was harvested 48 h later. We found that the mCherry-positive MP and BP cells located in the upper-IZ (presumably at the terminal-MP phase) expressed mVenus strongly, whereas the MP cells located in the lower-IZ (presumably at the early-MP phase) expressed mVenus weakly (Figure 1 H, H′, H″). These findings suggested that MP cells express distinct levels of Prdm8 protein depending on their position within the IZ.

On the basis of these characteristics of the expression pattern, we hypothesized that the precise expression of Prdm8 is critical for the regulation of the timing for the morphological transition in the post-mitotic cells during neural differentiation.

### Prdm8 regulates the morphological transition at MP phase

To investigate the role of Prdm8 in the developing neocortex, we carried out Prdm8 gain-of-function and loss-of-function experiments by using in utero electroporation. We transduced the E14.5 cortical VZ with a control (pCAG-IRES-Puro and pCAG-IRES-EGFP), an expression vector (pCAG-Prdm8 and pCAG-IRES-EGFP), or a knockdown vector (pPrdm8sh#629 and pCAG-IRES-EGFP). No significant difference was observed in the cell position between the control, gain-of-function or loss-of-function conditions at 48 h after electroporation, and EGFP-positive cells were mainly positioned in the lower-IZ or middle-IZ for in all cases (Figure S2A, B, C). However, we noticed that the gain-of-function condition increased the number of cells with MP morphology (Figure S2E, E′), whereas the Pdm8 loss-of-function condition increased the number of cells with the BP morphology (Figure S2F, F′). Therefore, we next focused on the role of Prdm8 in regulating the timing of the transition from MP to BP morphology at the late-MP and/or terminal-MP phases.

**Figure 2:**
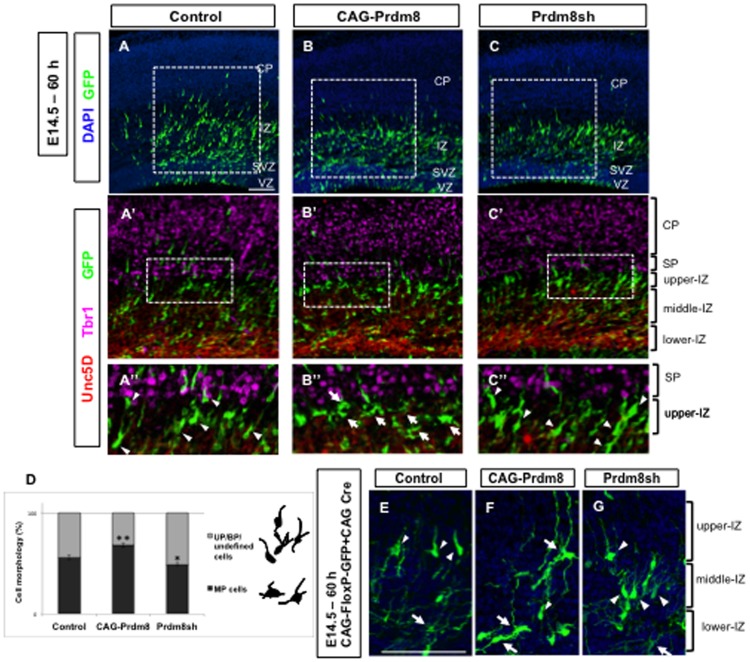
Prdm8 regulates the morphological changes at the MP phase. In utero electroporation of any one of control (pCAG-IRES-EGFP with pCAG-IRES-Puro; A), or Prdm8 gain-of-function (pCAG-IRES-EGFP with pCAG-Prdm8; B), or Prdm8 loss-of-function (pCAG-IRES-EGFP with pPrdm8sh#629; C) vectors were carried out at E14.5 and the brains were analyzed 60 h after the electroporation. Immunostaining was performed for Tbr1 (magenta) and Unc5D (red) to confirm the SP and middle-IZ, respectively (A′, B′, C′), and examination of the magnified images revealed that the majority of Prdm8 gain-of-function cells predominantly displayed MP morphology (B″, arrows), while Prdm8 loss-of-function cells predominantly displayed premature BP morphologies (C″, arrowheads) in any part of the IZ. Control manipulation contained both MP cells in lower- and middle-IZ, and BP cells in the upper-IZ (A′). Cell morphology was quantified to classify into two groups as “MP” shape and “UP/BP/undefined” shape (D). The number of counted cells was about 180 cells. Data represents the mean ± SD (n>3 slices from 3 individuals). *p<0.05, **p<0.01. Neocortical cells were labeled with a Cre-loxP expression plasmid, pCAG-FloxP-EGFP-N1 and pCAG-Cre, in the presence of either control (E), pCAG-Prdm8 (F), or pPrdm8sh (G) by using in utero electroporation to monitor the morphological difference clearly. The nuclei are stained with DAPI in A–C, E–G. Scale bars: 100 µm.

**Figure 3:**
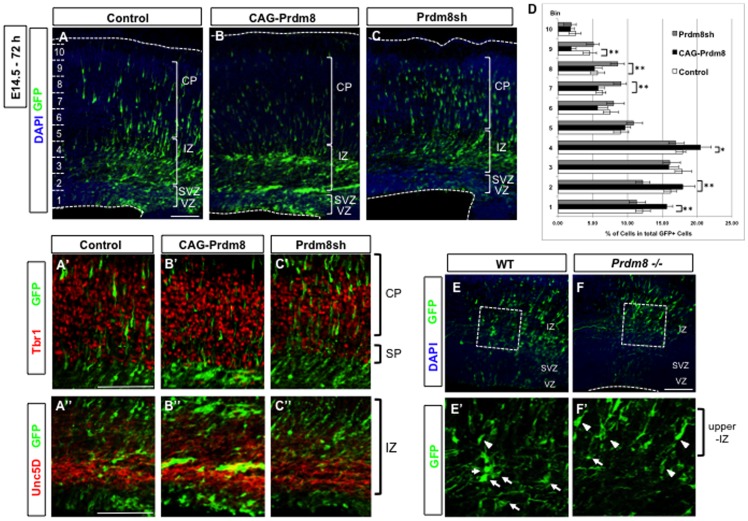
Prdm8 upregulation maintains the MP morphologies and Prdm8 downregulation displayed premature BP transition. In utero electroporation of any one of control (pCAG-IRES-EGFP with pCAG-IRES-Puro; A), or Prdm8 gain-of-function (pCAG-IRES-EGFP with pCAG-Prdm8; B), or Prdm8 loss-of-function (pCAG-IRES-EGFP with pPrdm8sh#629; C) vectors were carried out at E14.5 and brains were analyzed 72 h after the electroporation. Immunostaining with Tbr1 (red, A′, B′, C′) or Unc5D (red, A″, B″, C″) shows the organization of the SP and layer VI neurons or IZ, respectively. The majority of Prdm8 gain-of-function cells occur in different parts of the IZ (B, B′, B″) and show delayed migration into the CP, whereas the control cells are found inside the CP (A, A′). On the other hand, Prdm8 loss-of-function cells increased the EGFP-positive cells inside the CP (C, C′). The positions of EGFP-positive cells within the neocortex in control, CAG-Prdm8, and Prdm8sh plus EGFP plasmids in the brain were examined (D). The number of counted cells was about 203 cells. Data represents the mean ± SD (n>4 slices from 3 individuals); *p<0.05, **p<0.01. To further clarify the role of Prdm8, the WT (E) or *Prdm8−/−* (F) embryos were electroporated at E14.5 with the CAG-EGFP vector, and analyzed 54 h later. The majority of EGFP-positive cells possessed BP morphology (arrowheads) in *Prdm8*−/− brains (F′), whereas some EGFP-positive cells in the WT brains with MP morphology (arrows) (E′). The nuclei are stained with DAPI in A–C and E,F Scale bars: 100 µm.

For this purpose, we harvested the embryonic brains at 60 h (Figure 2), 72 h (Figure 3), and 120 h (Figures S2G–I), after the electroporation on E14.5. It has already been shown previously [36] that when the CAG-promoter-driven EGFP-expressing vector is electroporated in the E14.5 neocortex, EGFP-positive cells that express NeuroD1 in the emergence and accumulation zone of MP cells, are typically observed 36 h after electroporation, just above the VZ. The EGFP-positive cells further proceeded to the terminal-MP phase, after 60 h of electroporation. We found that after 60 h of electroporation, the overexpression of Prdm8 inhibited the MP-to-BP transition, but maintained MP morphology in the IZ (even just below the SP) (Figure 2B, B′, B″), although the vast majority of control cells started the transformation to the BP morphology in the same position (Figure 2A, A′, A″). On the other hand, the knockdown of Prdm8 caused to prematurely change the morphology from MP to BP (Figure 2C, C′, C″). Next, the percentage of cells that were MP and unipolar (UP)/BP/undefined morphology was determined (Figure 2D). We confirmed that Prdm8 overexpression led to a significant increase in the percentage of “MP cells” (68.3±2.3% vs. 56.1±2.6%; Prdm8 gain-of-function vs. control), and a decrease in the percentage of “UP/BP/undefined cells”. On the other hand, knockdown of Prdm8 resulted in trends opposite to those observed with Prdm8 overexpression, including an increase in the percentage of “UP/BP/undefined cells” (51.0±1.6% vs. 43.9±2.6%; Prdm8 loss-of-function vs. control). Furthermore, we labeled neocortical cells with a Cre-loxP clonal expression plasmid system, pCAG-FloxP-EGFP-N1 and pCAG-Cre in the presence of control, Prdm8 overexpression, or Prdm8 knockdown vector by using in utero electroporation to monitor the morphological differences more clearly. We also confirmed that the gain-of-function cells possessed preferentially the MP morphology (Figure 2F), whereas the Pdm8 loss-of-function cells possessed preferentially the BP morphology (Figure 2G). These results indicate the importance of expression level of Prdm8 in regulation of the timing of the morphological change from MP to BP in the IZ.

In addition, 72 h after this manipulation, the vast majority of Prdm8 gain-of-function cells (Figure 3B, B′, B″, D) still remained in the IZ and not within the CP, with MP morphologies (control; 31.9±3.2%, gain-of-function; 41.2±2.0%, loss-of-function; 31.1±2.2% MP cells by total number of EGFP positive cells, respectively). The control cells were located in both the CP and upper-IZ, and had BP morphology (Figure 3A, A′), and were also located in the lower-IZ or middle-IZ with MP morphology (Figure 3A, A″). On the other hand, few loss-of-function cells were found in the VZ/SVZ, but these cells were predominantly localized to the IZ or CP (Figure 3C, C′, C″). A similar phenotype was reproduced by the introduction of another knockdown vector, pPrdm8sh#2290, indicating that the effect of the knockdown of Prdm8 is not non-specific (data not shown). However, 120 h after the electroporation (P0), Prdm8 gain-of-function cells were found inside the CP (Figure S2H), thereby suggesting that the upregulation of Prdm8 caused a delay in migration and transition to BP morphology but did not permanently arrest the cells in the MP phase.

To further clarify the role of Prdm8 in the MP phase, we generated a *Prdm8−/−* mouse line (Figure S3) and investigated the timing of the morphological change in *Prdm8−/−* mouse brains by the introduction of a CAG-promoter-driven EGFP-expressing vector by using in utero electroporation at E14.5. EGFP-positive electroporated cells showed severe impairment in the timing of morphological change in *Prdm8−/−* (Figure 3F) when compared with WT cells (Figure 3E). The majority of EGFP-positive cells reached the upper-IZ, and preferentially possessed BP morphology at 54 h after electroporation in *Prdm8*−/− brains (Figure 3F, F′), whereas some EGFP-positive cells in the WT brains were still localized to the SVZ or lower-IZ and had MP morphology (Figure 3E, E′). Further, we confirmed that the percentage of “MP cells” was significantly decreased in *Prdm8−/−* (43.5±7.5% vs. 62.6±2.9%; *Prdm8−/−* vs. WT, n>3 from 3 litter mates), whereas the percentage of “UP/BP/undefined cells” was significantly increased in *Prdm8−/−*.

Taken together, these findings suggest that proper expression of Prdm8 in the MP phase is an important factor to regulate MP-to-BP transition at the appropriate timing during neural differentiation.

### Prdm8 alters layer formation in the neocortex

**Figure 4:**
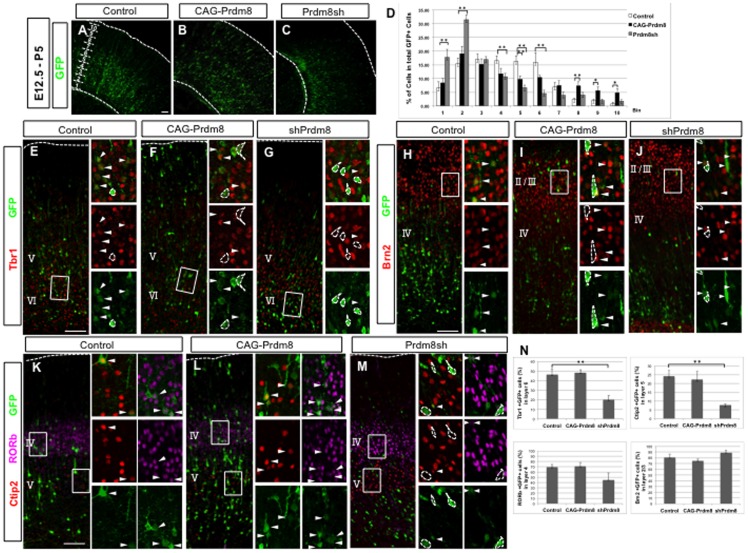
Prdm8 alters layer formation in the neocortex. In utero electroporation of any one of control (pCAG-IRES-EGFP with pCAG-IRES-Puro; A), or Prdm8 gain-of-function (pCAG-IRES-EGFP with pCAG-Prdm8; B), or Prdm8 loss-of-function (pCAG-IRES-EGFP with pPrdm8sh#629; C) vectors were carried out at E12.5, and the brains were analyzed at P5. The cortex was divided into 10 bins and the percentage of EGFP-positive cells were quantified (D). The distribution of EGFP-positive cells is significantly increased in the upper bins (bins8–10) and reduced in lower bins (bin5) in the case of the pCAG-Prdm8-electroporated brains, and significantly decreased in upper bins (bins4–6), and increased in lower bins (bins1, 2) in the pPrdm8sh-electroporated brains. The number of counted cells was about 137 cells. Data represents the mean ± SD (n = 6 slices from 3 individuals); *p<0.05, **p<0.01. High-power images showing that the molecular features of control, Prdm8 gain-of-function, or loss-of-function cells in the neocortex, stained with Tbr1 (E, F, G), Ctip2 (K, L, M), RORb (K, L, M) and Brn2 (H, I, J). The percentage of each layer marker-positive EGFP-positive cells located in each layer position was quantified (N). The number of counted cells was about 184 cells. Data represents the mean ± SD (n>4 slices from 4 individuals); **p<0.01. Scale bars: 100 µm (A,E).

We also examined Prdm8 gain-of-function and Prdm8 loss-of-function cells at P5, a stage at which neuronal migration is largely complete, after the electroporation at E12.5. When the control vector was used, EGFP positive neurons were found to be settled in layers II to VI of the neocortex at P5 (Figure 4A). In striking contrast, when the Prdm8 knockdown vector was used, EGFP- positive neurons were located primarily in the deep-layer (Figure 4C), whereas Prdm8 gain-of-function cells were located more superficially (Figure 4B). To quantify this difference, we analyzed the distribution of transfected cells in each bins (Figure 4D). In control cells, 28.2±4.8% of EGFP-labeled cells distributed into the upper layer (bins6–10), and 71.8±4.8% of EGFP-labeled cells distributed into the deep-layer (bins1–5), respectively. In contrast, 35.7±3.9% of Prdm8-overexpressing cells were located in the upper-layer, whereas 83.6±3.6% of Prdm8-knockdown cells were located in deep-layer.

The alteration in the laminar location did not clarify which of the following was true: (1) Prdm8 expression level simply affects the initiation of radial migration into the CP, thereby resulting in the alteration of cell distribution without changing neuronal identity, or (2) Prdm8 expression level could play a role in the determination of neuronal identity. To address this issue, at first we administered a pulse of EdU at E12.5 to label temporal cohorts of cortical neurons born at the early developmental stage, after the electroporation at E12.5 and analyzed them at P5. We found that the percentage of cells positive for both EdU and EGFP by the total number of EGFP-positive cells was significantly increased, and that these cells were located more deeply after the introduction of the Prdm8 knockdown vector (Figure S4). This suggested that the Prdm8 expression level at the embryonic stage affects the timing of neural differentiation. Next, the identity of the electroporated cells was analyzed by immunohistochemistry and quantified using markers specific to each layer, such as Brn2 (a marker for layer II/III [37], Figure 4H–J), RORb (a marker for layer IV [38], Figure 4K–M), Ctip2 (a marker for layer V [39], Figure 4K–M), and Tbr1 (a marker for layer VI [40], Figure 4E–G). The control EGFP-positive cells showed molecular features consistent with the laminar location (Figure 4E, H, K). In striking contrast, when the Prdm8 knockdown vector was used, EGFP-positive neurons located in the deep-layer showed a significant decrease in both Tbr1-positive and Ctip2-positive ratio (Figure 4G, M, N). We also observed some layer markers (Brn2, RORb, Ctip2, and Tbr1)-negative cells with neuronal morphology in the Prdm8 knockdown cells, located in the deep-layer. But, apoptotic cells were not increased significantly after the transfection of Prdm8 knockdown vector, excluding the possibility that some types of cells were selectively eliminated (data not shown). These results indicate that Prdm8 knockdown stimulated the timing of neural differentiation, and differentiated neurons located more deeply in the neocortex, however, those cells were inhibited to acquire molecular features consistent with laminar location. On the other hand, when the Prdm8 overexpression vector was used, EGFP-positive cells located in the upper-layer showed no significant difference in either Brn2-positive or RORb-positive ratio (Figure 4I, L, N), although the total number of Brn2-positive EGFP-positive cells was increased (25.6±4.4 cells vs. 19.2±1.6 cells; Prdm8 gain-of-function vs. control) after the overexpression of Prdm8 (data not shown).

These results suggest that the regulation of Prdm8 plays an important role both in the timing of neural differentiation and determination of neuronal identity.

### Prdm8 controls the expression level of guidance molecules

The ability of post-mitotic cells to simultaneously navigate through the developing cortex and acquire characteristic phenotypes is thus likely to depend on dynamic patterns of gene expression during the post-mitotic period (Figure 1K). However, the characterization of these cell-intrinsic, dynamic gene expression patterns remains incomplete, in particular, the molecular mechanisms controlling the MP phase have not yet been explained. Therefore, we performed the cell sorting of mVenus-positive and mVenus-negative cells from the E15.5 neocortex by FACS by taking advantage of a specific expression pattern in the *Prdm8*-mVenus mice (Figure S1H), and compared the gene expression profiles between these cells. We observed a marked upregulation of genes in the mVenus-positive cells, including *Reelin*, *NeuroD1*, and *Nhlh1*, the expression of which is restricted to the IZ (Table 1) [13], [41]. On the other hand, a marked downregulation of genes was confirmed in the mVenus-positive cells restricted to progenitors within the VZ or SVZ, which include *Pax6*, *Notch1*, *Hes1*, *Hes5*, *Sox2*, and *Tbr2* [8], [11], [35]. The microarray identified over 90 transcripts with two-fold higher expression in the mVenus-positive cells compared with that in the mVenus-negative cells at this stage (Table 2). Interestingly, these candidate genes, preferentially expressed at the late-MP and/or terminal-MP phases, included those involved in the signaling of guidance molecules, such as semaphorin signaling (*Plxnd1*, *Ebf3*, *Nrp2*, and *Sema3c*), ephrin signaling (*Epha6*), and slit signaling (*Slit3*) [42], thereby suggesting the possible involvement of these genes in the guidance of migrating MP cells.

**Table 1:** IZ or VZ/SVZ-expressed genes showing changes in microarray analyses from E15.5 *Prdm8*-mVenus mouse neocortex.

Gene Symbol	Fold Change (mVenus+/−)	Gene Title	Genbank
**Intermediate zone-expreesed genes**			
*Reln*	4.95	reelin	U24703
*Neurod1*	2.55	neurogenic differentiation 1	BC094611
*Prdm8*	2.43	PR domain containing 8	BC141020
*Nhlh1*	2.03	nescient helix loop helix 1	M97506
**Ventricular zone and sub ventricular zone-expressed genes**			
*Sox2*	−4.81	SRY-box containing gene 2	BC057574
*Notch1*	−4.45	Notch gene homolog 1 (Drosophila)	BC138441
*Eomes*	−3.13	eomesodermin homolog (Xenopus laevis)	BC094319
*Hes1*	−3.10	hairy and enhancer of split 1 (Drosophila)	BC018375
*Pax6*	−2.80	paired box gene 6	BC036957

**Table 2:** Microarray expression analyses of mVenus-positive versus mVenus-negative cells from E15.5 *Prdm8*-mVenus mouse neocortex.

Gene Symbol	Fold Change (mVenus+/−)	Gene Title	Genbank
*Hbb-y*	13.80	hemoglobin Y, beta-like embryonic chain	BC057014
*A930038C07Rik*	8.64	RIKEN cDNA A930038C07 gene	BC047154
*Calb2*	8.62	calbindin 2	BC017646
*Grp*	7.30	gastrin releasing peptide	BC024515
*Fam163a*	6.72	family with sequence similarity 163, member A	BC116972
*Ifi203*	6.37	interferon activated gene 203	AF022371
*Crym*	6.22	crystallin, mu	AF039391
*Rgs4*	5.48	regulator of G-protein signaling 4	DQ346660
*Crabp1*	4.98	cellular retinoic acid binding protein I	X15789
*Reln*	4.95	reelin	U24703
*Trp73*	4.65	transformation related protein 73	BC066045
*Gabra2*	4.44	gamma-aminobutyric acid (GABA) A receptor, subunit alpha 2	M86567
*Rerg*	4.23	RAS-like, estrogen-regulated, growth-inhibitor	BC026463
*Nr4a3*	3.91	nuclear receptor subfamily 4, group A, member 3	BC068150
*Nhlh2*	3.89	nescient helix loop helix 2	BC058413
*Slit3*	3.85	slit homolog 3 (Drosophila)	BC150780
*5330417C22Rik*	3.84	RIKEN cDNA 5330417C22 gene	BC051424
*Ppp2r2c*	3.82	protein phosphatase 2 (formerly 2A), regulatory subunit B (PR 52), gamma isoform	BC059811
*Rit2*	3.82	Ras-like without CAAX 2	BC018267
*S100a10*	3.76	S100 calcium binding protein A10 (calpactin)	BC025044
*Mab21l1*	3.58	mab-21-like 1 (C. elegans)	AF228913
*Cacna2d2*	3.52	calcium channel, voltage-dependent, alpha 2/delta subunit 2	BC158058
*Ebf3*	3.48	early B-cell factor 3	BC067018
*Car10*	3.42	carbonic anhydrase 10	AB080741
*Tuft1*	3.38	tuftelin 1	BC019213
*Mical2*	3.35	microtubule associated monoxygenase, calponin and LIM domain containing 2	AK220353
*Cpne4*	3.31	copine IV	BC043087
*Tacr3*	3.29	tachykinin receptor 3	BC066845
*St6galnac5*	3.26	ST6 (alpha-N-acetyl-neuraminyl-2,3-beta-galactosyl-1,3)-N-acetylgalactosaminide alpha-2,6-sialyltransferase 5	AB028840
*Epha6*	3.26	Eph receptor A6	U58332
*Hcn1*	3.23	hyperpolarization-activated, cyclic nucleotide-gated K+ 1	AF028737
*Cryab|Hspb2*	3.23	crystallin, alpha B | heat shock protein 2	BC094033
*Olfml2b*	3.22	olfactomedin-like 2B	BC025654
*Rcan2*	3.21	regulator of calcineurin 2	BC049096
*Pcdh20*	3.18	protocadherin 20	BC079605
*Plxnd1*	3.12	plexin D1	AY688678
*Lhx5*	3.00	LIM homeobox protein 5	U61155
*Pde1a*	2.99	phosphodiesterase 1A, calmodulin-dependent	BC090628
*Slc7a8*	2.99	solute carrier family 7 (cationic amino acid transporter, y+ system), member 8	BC059004
*B830028B13Rik*	2.97	RIKEN cDNA B830028B13 gene	BC158077
*Cacna2d3*	2.96	calcium channel, voltage-dependent, alpha2/delta subunit 3	CR457444
*Fam70a*	2.94	family with sequence similarity 70, member A	BC062956
*Nrp2*	2.94	neuropilin 2	AF483506
*Zfp385b*	2.92	zinc finger protein 385B	BC132352
*Pappa2*	2.84	pappalysin 2	BC104644
*Clstn2*	2.84	calsyntenin 2	BC063058
*Cck*	2.81	cholecystokinin	BC028487
*Stxbp5l*	2.81	syntaxin binding protein 5-like	AY542324
*Sphkap*	2.80	SPHK1 interactor, AKAP domain containing	BC069832
*Gabbr2*	2.78	gamma-aminobutyric acid (GABA) B receptor, 2	AF095784
*Lhx1*	2.77	LIM homeobox protein 1	BC092374
*Chrna7*	2.77	cholinergic receptor, nicotinic, alpha polypeptide 7	L37663
*Mctp1*	2.77	multiple C2 domains, transmembrane 1	BC030005
*Mgat4c*	2.73	mannosyl (alpha-1,3-)-glycoprotein beta-1,4-N-acetylglucosaminyltransferase, isozyme C (putative)	BC046987
*Nrip3*	2.73	nuclear receptor interacting protein 3	BC072641
*1810041L15Rik*	2.70	RIKEN cDNA 1810041L15 gene	BC062953
*Lhx9*	2.68	LIM homeobox protein 9	BC072623
*Ache*	2.66	acetylcholinesterase	BC046327
*Slc4a4*	2.63	solute carrier family 4 (anion exchanger), member 4	AF141934
*Nrxn1*	2.62	neurexin I	BC047146
*Shisa6*	2.62	shisa homolog 6 (Xenopus laevis)	NM207386
*Opcml*	2.60	opioid binding protein/cell adhesion molecule-like	BC076581
*Spock2*	2.58	sparc/osteonectin, cwcv and kazal-like domains proteoglycan 2	BC057324
*Dnajc6*	2.57	DnaJ (Hsp40) homolog, subfamily C, member 6	BC060734
*Chrm3*	2.56	cholinergic receptor, muscarinic 3, cardiac	BC129892
*Fabp3*	2.55	fatty acid binding protein 3, muscle and heart	BC089542
*Timp3*	2.54	tissue inhibitor of metalloproteinase 3	BC014713
*Adora1*	2.52	adenosine A1 receptor	BC079624
*P2rx5*	2.50	purinergic receptor P2X, ligand-gated ion channel, 5	AF333331
*Sv2b*	2.49	synaptic vesicle glycoprotein 2 b	BC060224
*Fosl2*	2.49	fos-like antigen 2	BC065131
*Camk2b*	2.48	calcium/calmodulin-dependent protein kinase II, beta	BC080273
*Tbata*	2.48	thymus, brain and testes associated	AF257502
*Cdkn1a*	2.44	cyclin-dependent kinase inhibitor 1A (P21)	BC002043
*Prdm8*	2.43	PR domain containing 8	BC141020
*Barhl2*	2.43	BarH-like 2 (Drosophila)	BC078444
*Wbscr17*	2.43	Williams-Beuren syndrome chromosome region 17 homolog (human)	BC158110
*Zdhhc23*	2.42	zinc finger, DHHC domain containing 23	BC139052
*Edil3*	2.42	EGF-like repeats and discoidin I-like domains 3	BC056386

The genes with expression levels more than 2.4 fold higher in *Prdm8*-mVenus positive cells compared to mVenus negative cells are listed. From this analysis (n = 2), we have identified several genes showing specific expression within middle-IZ and/or upper-IZ.

**Figure 5:**
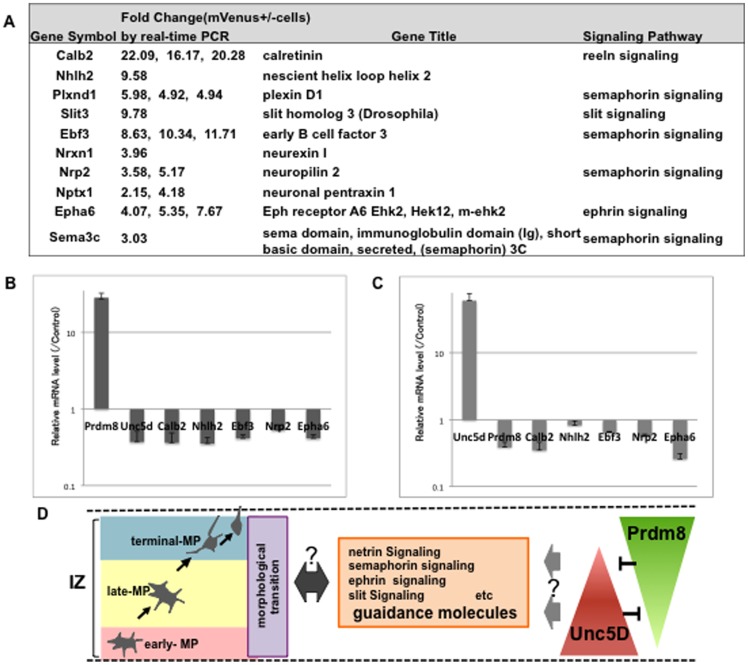
Candidate genes, preferentially expressed in the late-MP and/or terminal-MP phases. Ten candidate genes were selected by the validation of DNA microarray data (A) (n = 2). Quantitative real-time PCR data shows the expression level of some candidate genes was suppressed by the introduction of pCAG-Prdm8 (B) (n = 4) or pCAG-Unc5D (C) (n = 3). Schematic drawing of a working hypothesis of this study (D).

Further, we validated the microarray data using publicly available in situ hybridization datasets from Allen Brain Atlas, and confirmed whether the listed genes were expressed in the telencephalon at embryonic stages, and the expression levels in mVenus-positive and mVenus-negative cells were validated to check the reproducibility by quantitative real-time PCR (Figure 5A). We finally selected 10 candidate genes that were preferentially expressed in the late-MP and/or terminal-MP phases, similar to Prdm8 (Figure 5A).

Next, to test the correlation between Prdm8 and these candidate genes, either control (pCAG-IRES-Puro and pCAG-IRES-EGFP), or Prdm8 expression vector (pCAG-Prdm8 and pCAG-IRES-EGFP) was introduced into primary dissociated neocortical cells on E14.5 by the *in vitro* electroporation system. After electroporation, cells were cultured in neurosphere media for 2 days and then EGFP-positive cells (usually 15–20% of the cultures) were isolated by FACS for further analysis. Prdm8 overexpression in neocortical cells significantly suppressed the expression of Calb2, Nhlh2, Ebf3, Nrp2, and Epha6 (Figure 5B). Furthermore, the expression of Unc5D was also decreased more than 2-fold by the introduction of the Prdm8 expression vector. On the other hand, we also examined the introduction of the Unc5D expression vector (pCAG-Unc5D and pCAG-IRES-EGFP) in the same experimental system, and we found that Unc5D overexpression also significantly suppressed the expression of Calb2, Ebf3, Nrp2, and Epha6 (Figure 5C). Interestingly, we observed that Prdm8 expression was significantly suppressed by the overexpression of Unc5D.

Thus, we propose a working hypothesis that Prdm8 controls the transition from MP to BP morphology through the balance of expression level of some guidance molecules in the IZ (Figure 5D), and that this regulation of the MP phase plays an important role in proper neocortical lamination.

## Discussion

The finding of this study indicated that Prdm8 is one of the important factors in regulating the MP phase during the precise transition from MP to BP morphology. Prdm8 shows a highly specific expression patterning in the embryonic neocortex: no expression in the VZ and SVZ, weak expression in the lower-IZ, and very strong expression in the middle-IZ and upper-IZ. This pattern suggests that Prdm8 has distinct functions in each step of the development of post-mitotic MP cells during which they cause dynamic morphological changes in the IZ.

Precise control of neuronal migration is essential for the development of the neocortex [6]. Post-mitotic cells within the IZ transiently have been found to assume a characteristic MP morphology [13]–[24]. Subsequently, the MP-to-BP transition occurs before the cells enter the CP [7], [15]. A considerable amount of research has been done on the mechanism responsible for transformation from a MP migration mode to a locomotion mode. Several molecules, such as Cdk5 [43], CRIMP2 [44], filamin A [45], and SCG10/Stathmin-2 [46], have been reported to be involved in this MP-to-BP transition. In addition, it has been suggested that MP phase is vulnerable and is disrupted in several disorders of neocortical development [6]. Indeed, several studies revealed that the mutations in the genes encoding LIS1 [47] and DCX [48] in humans cause distinct neuronal migration disorders. However, significance of this MP phase for the establishment of mature cortical cytoarchitecture remains largely unknown. Especially, the molecular mechanism underlying MP migration for the regulation of each step at the MP phase in the IZ is far from understood.

Recent studies have shown that many transcription factors play key roles as molecular switches in regulating each step of the neural precursors located in VZ, SVZ, and IZ, while they proceed with their cell-intrinsic program [8]. Several authors have characterized these cell-intrinsic, and dynamic gene expression patterns has been tried to proceed by several approaches. For example, the transcriptional profiling of microdissected samples derived from developing brain [49]–[51], and more recently, the combined application of cell sorting from acutely dissociated brain and subsequent transcriptional profiling, have been performed [52], [53]. In this study, taking advantage of a specific expression pattern of *Prdm8*-mVenus mouse line, we analyzed the detailed expression profile of the sorted mVenus-positive cells. We identified the candidate genes, predominantly utilized at the late-MP and/or terminal-MP phase, which include those encoding for semaphorin, ephrin, and slit signaling molecules. These kinds of guidance molecules have been implicated in the regulation of a variety of cellular functions, including cell migration, morphological change, and axon guidance [54]–[56]. However, the role of the guidance molecules in regulating the MP phase remains largely unknown. A recent study has demonstrated that Unc5D is expressed by MP cells [5], [13], [57]. More recently, FoxG1 downregulation in the IZ has been reported to be crucial for MP cells to initiate Unc5D expression and to rapidly proceed from early-MP to late-MP phase [13]. In the present study, we used a combination of gain-of-function and loss-of function experiments by in utero electroporation, and found evidence that Prdm8 prolonged the duration of MP phase. Hence, we infer that Prdm8 critically regulates the timing of the morphological transition from MP to BP at appropriate timing during differentiation. In addition, interestingly, the expression level of guidance molecules such as Ebf3, Nrp2, and Epha6 was significantly suppressed by the overexpression of Prdm8. Overexpression of Prdm8 also suppressed Unc5D mRNA expression, and vise versa. This interaction among Prdm8, Unc5D, and candidate genes may be important to regulate the migration and/or morphological transition at MP phase.

Our data also suggest that the timing of the transformation from MP to BP morphology at MP phase was highly correlated with the formation of the neocortical laminar during development. Other studies, including our previous study, have shown that the Prdm8 expression at the postnatal stage is mainly localized to the upper-layer neocortex [29], [30]. In addition, a genome-wide analysis by chromatin immunoprecipitation followed by deep sequencing (ChIP-seq) revealed that Prdm8 could bind to some transcription factors that correlated with the fate determination of each specific layer neurons, including *Satb2*, *Unc5D*, and *Cux1* (data not shown). These data support the idea that precise expression of Prdm8 is critical for regulation of the timing for morphological transition, and that it plays an important role in generation of distinct neocortical lamina precisely. A recent study has demonstrated that the dynamic variation of FoxG1 expression in the IZ is essential for layer formation of neocortex, and that fate determination of pyramidal neurons remains labile at least up to the early-MP phase [13]. Thus, Prdm8 expression restricted in a spatial and temporal manner during the late-MP and terminal-MP phases plays an important role as a molecular switch in the regulation of neocortical cytoarchitecture.

In summary, we found that Prdm8 can influence the timing of neural differentiation by controlling the precise transition from the MP to BP morphology at the MP phase. In other words, less time spent in the IZ as a result of Prdm8 downregulation will result in proceeding to differentiate prematurely, but differentiated neurons lose their precise laminar identity. It will be interesting to analyze whether the guidance molecules, identified as candidate genes, contribute to the action of Prdm8, which in turn determines the cell fate during the MP phase.

## Supporting Information

Figure S1 **The mVenus is strongly expressed in the post-mitotic cells of the developing neocortex of mice with** ***Prdm8*** **reporter expression.** Strategy to detect Prdm8 expression in the *Prdm8*-mVenus mice line (A). Immunostaining reveals that Prdm8 mVenus expression pattern in transgenic mouse is similar to the immunostaining pattern of obtained with anti-Prdm8 antibody at E15.5 (B, B′, B″). This pattern is specifically expressed in the post-mitotic neurons, which were co-labeled with not PCNA (red, C), but Tuj1 (red, D, D′). The mVenus was strongly expressed at E14.5 (E), but gradually decreased after E17.5 (F,G). The cell sorting scheme was used for DNA microarray analysis (H). The nuclei are stained with DAPI in B, E–G. Scale bars: 100 µm.
(TIFF)

Figure S2 **Prdm8 upregulation maintains MP morphologies transiently, but the cells enter the CP later.** In utero electroporation of any one of control (pCAG-IRES-EGFP with pCAG-IRES-Puro; A), or Prdm8 gain-of-function (pCAG-IRES-EGFP with pCAG-Prdm8; B), or Prdm8 loss-of-function (pCAG-IRES-EGFP with pPrdm8sh#629; C) vectors were carried out at E14.5 and the brains were analyzed 48 h (A–C) and 120 h (G–I) after the electroporation. In utero electroporation of Cre-loxP clonal expression plasmid system was performed by using pCAG-FloxP-EGFP-N1 and pCAG-Cre (D–F). Magnified images revealed that the majority of Prdm8 gain-of-function cells predominantly displayed MP morphologies (arrows: E′) and Prdm8 loss-of-function cells predominantly displayed BP morphologies (arrowheads; F′), whereas control manipulation contained both MP and BP cells in the IZ (D′). The nuclei are stained with DAPI in A–C, D–F, and G–I. Scale bars: 100 µm.
(TIFF)

Figure S3 **Generation and validation of the** ***Prdm8*** **null allele.** The targeting vector for the *Prdm8* genomic locus was constructed by using double positive selection cassettes. At first, exon 2 (E2) was replaced by a loxP(solid triangle)-flanked PGK-driven Neo gene (A). Second, the region downstream of exon 4 to exon 5 (E4–E5) of Prdm8 was replaced by a FRT(empty triangle)-flanked PGK-Neo gene (B). This targeted allele with *loxP-Prdm8-loxP-FRT-Neo-FRT* was removed by crossing with *VASA*-Cre mouse line (C). PCR genotyping for the variants of Prdm8 mutant loci was carried out using the primer sets, p1 and p2. Western blot analysis showing Prdm8 protein levels in the P13 brains of the indicated genotypes (D).
(TIFF)

Figure S4 **Prdm8 downregulation increased early born neurons.** Pulse of EdU labeling was performed at E12.5, after in utero electroporation of any one of control (pCAG-IRES-EGFP with pCAG-IRES-Puro; A), or Prdm8 loss-of-function (pPrdm8sh#629 with pCAG-Prdm8; B) at E12.5, and analyzed at P5. Magnified images revealed that EdU-positive cells predominantly localized more deeply by the Prdm8 downregulation (A′, B′). Cells positive for both EdU and EGFP by the total number of EGFP-positive were quantified (C). Data represents the mean ± SD (n>3 slices from 2 individuals); *p<0.05.
(TIFF)
