# Supplementary figures and images for: Prdm8 Regulates the Morphological Transition at Multipolar Phase during Neocortical Development
Source: PLoS One. 2014 Jan 29;9(1):e86356. doi: 10.1371/journal.pone.0086356 (PMC3906029; doi:10.1371/journal.pone.0086356)

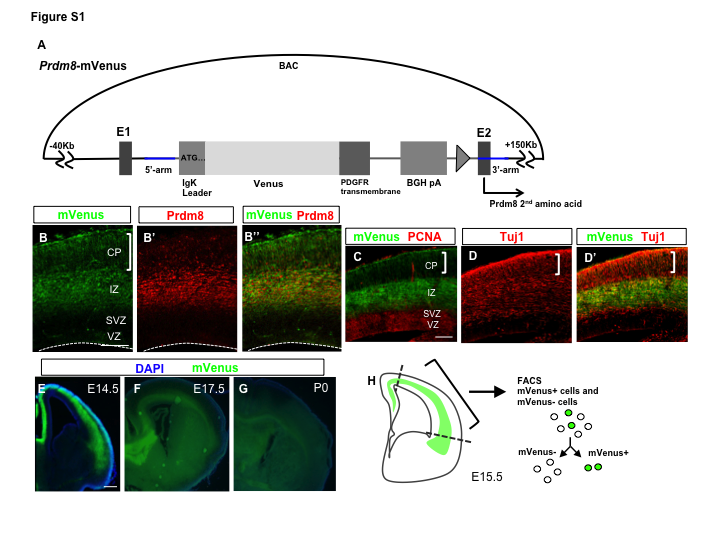

Supplement: Figure S1 — The mVenus is strongly expressed in the post-mitotic cells of the developing neocortex of mice with Prdm8 reporter expression. Strategy to detect Prdm8 expression in the Prdm8-mVenus mice line (A). Immunostaining reveals that Prdm8 mVenus expression pattern in transgenic mouse is similar to the immunostaining pattern of obtained with anti-Prdm8 antibody at E15.5 (B, B′, B″). This pattern is specifically expressed in the post-mitotic neurons, which were co-labeled with not PCNA (red, C), but Tuj1 (red, D, D′). The mVenus was strongly expressed at E14.5 (E), but gradually decreased after E17.5 (F,G). The cell sorting scheme was used for DNA microarray analysis (H). The nuclei are stained with DAPI in B, E–G. Scale bars: 100 µm. (TIFF) [file pone.0086356.s001.tiff]

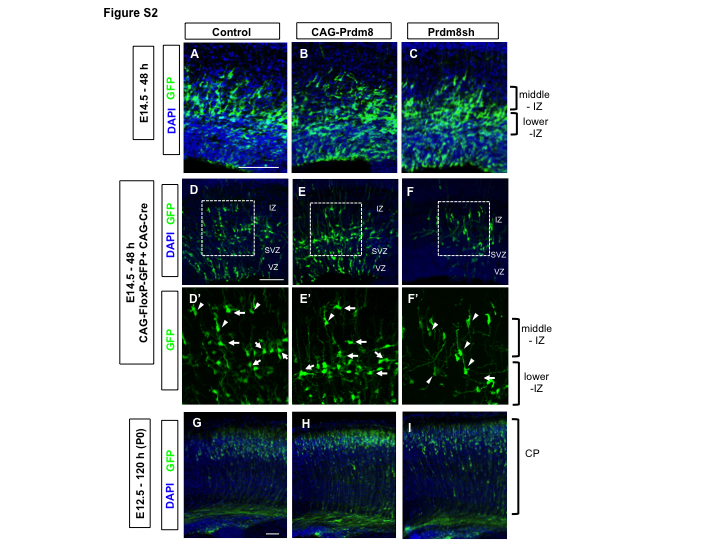

Supplement: Figure S2 — Prdm8 upregulation maintains MP morphologies transiently, but the cells enter the CP later. In utero electroporation of any one of control (pCAG-IRES-EGFP with pCAG-IRES-Puro; A), or Prdm8 gain-of-function (pCAG-IRES-EGFP with pCAG-Prdm8; B), or Prdm8 loss-of-function (pCAG-IRES-EGFP with pPrdm8sh#629; C) vectors were carried out at E14.5 and the brains were analyzed 48 h (A–C) and 120 h (G–I) after the electroporation. In utero electroporation of Cre-loxP clonal expression plasmid system was performed by using pCAG-FloxP-EGFP-N1 and pCAG-Cre (D–F). Magnified images revealed that the majority of Prdm8 gain-of-function cells predominantly displayed MP morphologies (arrows: E′) and Prdm8 loss-of-function cells predominantly displayed BP morphologies (arrowheads; F′), whereas control manipulation contained both MP and BP cells in the IZ (D′). The nuclei are stained with DAPI in A–C, D–F, and G–I. Scale bars: 100 µm. (TIFF) [file pone.0086356.s002.tiff]

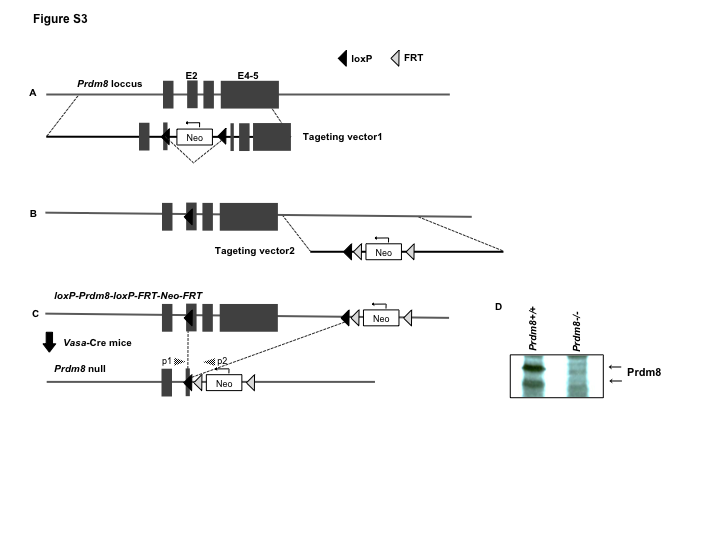

Supplement: Figure S3 — Generation and validation of the Prdm8 null allele. The targeting vector for the Prdm8 genomic locus was constructed by using double positive selection cassettes. At first, exon 2 (E2) was replaced by a loxP(solid triangle)-flanked PGK-driven Neo gene (A). Second, the region downstream of exon 4 to exon 5 (E4–E5) of Prdm8 was replaced by a FRT(empty triangle)-flanked PGK-Neo gene (B). This targeted allele with loxP-Prdm8-loxP-FRT-Neo-FRT was removed by crossing with VASA-Cre mouse line (C). PCR genotyping for the variants of Prdm8 mutant loci was carried out using the primer sets, p1 and p2. Western blot analysis showing Prdm8 protein levels in the P13 brains of the indicated genotypes (D). (TIFF) [file pone.0086356.s003.tiff]

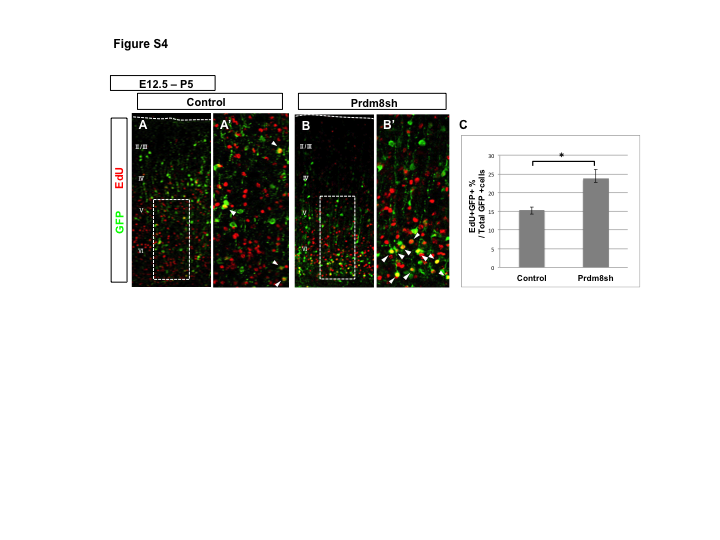

Supplement: Figure S4 — Prdm8 downregulation increased early born neurons. Pulse of EdU labeling was performed at E12.5, after in utero electroporation of any one of control (pCAG-IRES-EGFP with pCAG-IRES-Puro; A), or Prdm8 loss-of-function (pPrdm8sh#629 with pCAG-Prdm8; B) at E12.5, and analyzed at P5. Magnified images revealed that EdU-positive cells predominantly localized more deeply by the Prdm8 downregulation (A′, B′). Cells positive for both EdU and EGFP by the total number of EGFP-positive were quantified (C). Data represents the mean ± SD (n>3 slices from 2 individuals); *p<0.05. (TIFF) [file pone.0086356.s004.tiff]
